# An evaluation of respiratory administration of measles vaccine for prevention of acute lower respiratory infections in children

**DOI:** 10.1186/1471-2458-11-S3-S31

**Published:** 2011-04-13

**Authors:** Daisy Higginson, Evropi Theodoratou, Harish Nair, Tanvir Huda, Lina Zgaga, Suresh S Jadhav, Saad B Omer, Igor Rudan, Harry Campbell

**Affiliations:** 1 Centre for Population Health Sciences, Global Health Academy, The University of Edinburgh, UK; 2 Public Health Foundation of India, New Delhi, India; 3 International Centre for Diarrhoeal Disease Research, Bangladesh (ICDDR,B), Dhaka, Bangladesh; 4 Serum Institute of India Limited, Pune, India; 5 Emory University, Rollins School of Public Health, Atlanta, GA, USA; 6 Croatian Centre for Global Health, University of Split Medical School, Croatia

## Abstract

**Background:**

Measles was responsible for an estimated 100,000 deaths worldwide in 2008. Despite being a vaccine-preventable disease, measles remains a major cause of morbidity and mortality in young children. Although a safe and effective injectable measles vaccine has been available for over 50 years it has not been possible to achieve the uniformly high levels of coverage (required to achieve measles eradication) in most parts of the developing world. Aerosolised measles vaccines are now under development with the hope of challenging the delivery factors currently limiting the coverage of the existing vaccine.

**Methods:**

We used a modified CHNRI methodology for setting priorities in health research investments to assess the strengths and weaknesses of this emerging intervention to decrease the burden of childhood pneumonia. This was done in two stages. In Stage I, we systematically reviewed the literature related to emerging aerosol vaccines against measles relevant to several criteria of interest. Although there are a number of different aerosol vaccine approaches under development, for the purpose of this exercise, all were considered as one intervention. The criteria of interest were: answerability; cost of development, production and implementation; efficacy and effectiveness; deliverability, affordability and sustainability; maximum potential impact on disease burden reduction; acceptability to the end users and health workers; and effect on equity. In Stage II, we conducted an expert opinion exercise by inviting 20 experts (leading basic scientists, international public health researchers, international policy makers and representatives of pharmaceutical companies). The policy makers and industry representatives accepted our invitation on the condition of anonymity, due to the sensitive nature of their involvement in such exercises. They answered questions from the CHNRI framework and their “collective optimism” towards each criterion was documented on a scale from 0 to 100%.

**Results:**

The panel of experts expressed mixed feelings about an aerosol measles vaccine. The group expressed low levels of optimism regarding the criteria of likelihood of efficacy and low cost of development (scores around 50%); moderate levels of optimism regarding answerability, low cost of production, low cost of implementation and affordability (score around 60%); and high levels of optimism regarding deliverability, impact on equity and acceptability to health workers and end-users (scores over 80%). Finally, the experts felt that this intervention will have a modest but nevertheless important impact on reduction of burden of disease due to childhood pneumonia (median: 5%, interquartile range 1-15%, minimum 0%, maximum 45%).

**Conclusion:**

Aerosol measles vaccine is at an advanced stage of development, with evidence of good immunogenicity. This new intervention will be presented as a feasible candidate strategy in the campaign for global elimination of measles. It also presents an unique opportunity to decrease the overall burden of disease due to severe pneumonia in young children.

## Background

Despite the availability of an effective vaccine, the global burden of disease due to measles continues to be high in young children. Although the coverage of the first dose of measles vaccine improved dramatically in the twenty first century, it is estimated that in the year 2008 approximately 100,000 deaths in children aged less than 5 years was attributable to measles [1,2]. Failure to achieve universal coverage for at least one dose of measles vaccine remains the key reason for these deaths. Pneumonia is one of the most common fatal complications of measles [3]. In 2008, all World Health Organization (WHO) member states reaffirmed their commitment to reduce the global measles mortality to less than 75,000 deaths by 2010 (i.e. 90% reduction in measles mortality compared to the year 2000) [4]. This would in turn contribute to an overall reduction in burden of disease due to childhood pneumonia.

The debate regarding the feasibility of achieving a measles free world has been ongoing for the last two decades. The meeting of the International Task Force for Disease Eradication in June 2009 evaluated the available evidence for the potential eradicability of measles and concluded with greater confidence (than at the previous meeting in 2002) that “measles eradication is biologically possible using available tools…Research to discover new tools or to improve existing ones is needed to strengthen the arsenal against this contagious disease. Any practical breakthrough in ways to mitigate any of the current requirements to inject, provide 2 doses, refrigerate measles vaccine, and to improve efficacy in young infants, would be a major contribution to measles eradication” [5]. Two of the major components of the comprehensive strategy for measles eradication were achieving and maintaining high coverage (>90%) with the routinely scheduled first dose of measles-containing vaccine (MCV1) among children aged 1 year and to ensure that all children receive a second opportunity for measles immunization (either through a second routine dose or through periodic supplementary immunization activities (SIAs)). However, to implement these strategies in the field, a more simplified and effective delivery method would need to be developed.

The injectable measles vaccine received its licensure in 1963 and since then worldwide vaccination programmes have been employed in an attempt to control the disease [6]. The existing vaccine has proved extremely efficacious in the prevention of the disease. Between 2000 and 2007 global measles mortality declined by 74 percent with a 10 percent overall increase in vaccine coverage (estimated worldwide coverage of the first dose of measles vaccine in 2007 was approximately 82 percent) [1]. However, this coverage is not uniform- there are wide disparities in coverage among countries and pockets of poor coverage remain within countries with overall coverage above 80 percent. In 2007, MCV1 coverage in WHO Africa and South East Asia regions was 74% and 73% respectively [7] – two regions particularly vulnerable to disease out-breaks and which contribute to a substantial proportion of the global disease burden of measles. WHO has identified 47 priority countries which account for the highest percentage of measles deaths and have been made the focus of a comprehensive strategy for a targeted disease reduction [4]. As a result, researchers are exploring more effective ways to achieve universal coverage of measles vaccine amongst the poorer sections of the society in low and middle income countries. One of the strategies under consideration to improve delivery of the existing measles vaccine is needle free administration of the vaccine. Such a delivery mechanism could help achieve universal coverage of the vaccine in the hard to reach areas in resource poor settings[8].

In 1983, Albert Sabin described his vision of creating an aerosolized measles vaccine [9]. His concept was to create a vaccine that was simple, safe, cost-efficient and could be easily implemented into mass vaccination campaigns. The initial vaccine developed by him was piloted in Mexico and data from the limited serological and field studies demonstrated the safety and immunogenicity of the vaccine in infants and children [10]. Following this, more than 4 million children throughout Mexico were vaccinated against measles using the aerosol route of administration during mass campaigns [11]. However, the success was limited as the aerosol vaccine (effective in children aged 1 year and above) was never tested or incoporated into routine immunization programmes. In 2002 the Measles Aerosol Project (MAP) was established to develop at least one method for respiratory delivery of currently licensed measles vaccines [12]. In addition to MAP, there are a number of other research efforts to develop and evaluate an aerosol measles vaccine.

We aimed to review the existing literature, outlining the progress of the emerging aerosol measles vaccines at all stages of development; present the evidence regarding key issues surrounding aerosolized measles vaccine and assess the level of collective optimism of international experts over its priority status for receiving investment support as a strategy to decrease pneumonia burden in children. The paper is presented as part of a series of papers, each in turn focusing on different emerging vaccines and other interventions against pneumonia.

## Methods

We used a modified Child Health and Nutrition Research Initiative (CHNRI) methodology for setting priorities in health research investments. The methodology has been described in great detail [13-17] and implemented in a variety of settings [18-23]. Briefly, the method uses a set of pre-defined criteria and collects expert opinion of all stakeholders on the risks and benefits associated with investing in existing and / or new interventions.

### CHNRI exercise – stage I: Identification and selection of studies

**Figure 1:**
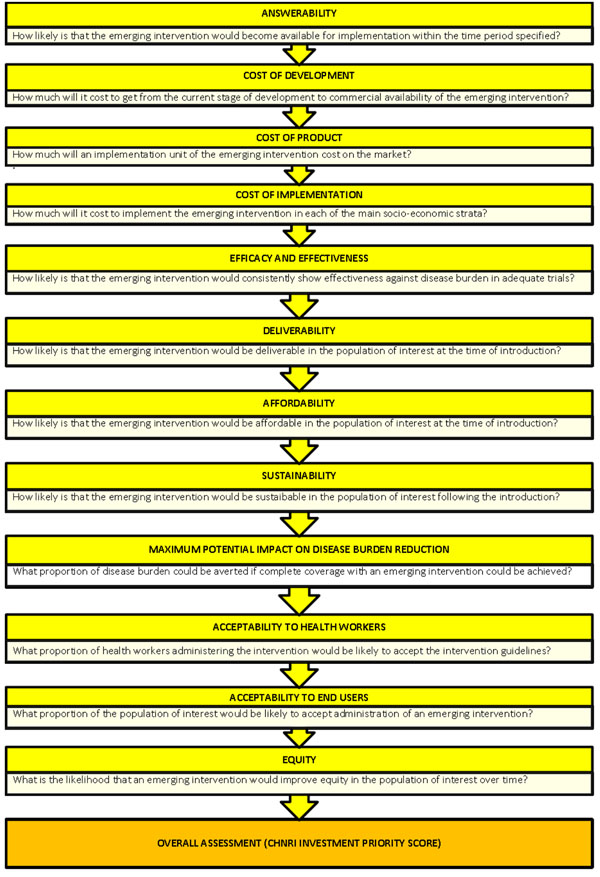
**A summary of Stage I of the CHNRI process of evaluation of an emerging intervention (a systematic review of the key CHNRI criteria)**. CHNRI- Child Health and Nutrition Research Initiative

We applied the CHNRI method to estimate the potential impact of the aerosolized measles vaccines. We conducted a systematic literature review using the following criteria: answerability, cost of development, cost of product, cost of implementation, efficacy and effectiveness, deliverability, affordability, sustainability, maximum potential impact on disease burden reduction, acceptability to health workers, acceptability to end users and equity [13] (Figure 1). Details about the search strategies are presented in Supplementary table 1 in Additional file 1. The search was limited to Ovid MEDLINE, Embase, Global Health, Web of Science, LILACS, IndMed, and grey literature (SIGLE) databases from January 1994 to May 2009. (Although we updated the search in January 2011, for Stage II of this exercise, the experts were presented only with a summary of the literature from 1994 to May 2009). No language or publication restrictions were applied. In order to ensure completeness, we also conducted hand searching of online journals, scanned the reference list of identified citations, and perused literature available on the websites of pharmaceutical companies and international agencies (GAVI and WHO).

We used a pre-determined eligibility criteria for screening and including identified studies. In particular, we included studies on aerosolized measles vaccine derived from either Edmonston-Zagreb or Schwarz vaccine virus strains in children aged less than15 years;studies assessing other novel interventions against measles; and studies addressing questions on answerability, effectiveness, deliverability, disease burden reduction or equity. We excluded studies reporting existing measles interventions; use of measles vaccine in the adult population; and not directly challenging the impact of vaccines.

### CHNRI exercise – stage II

**Figure 2:**
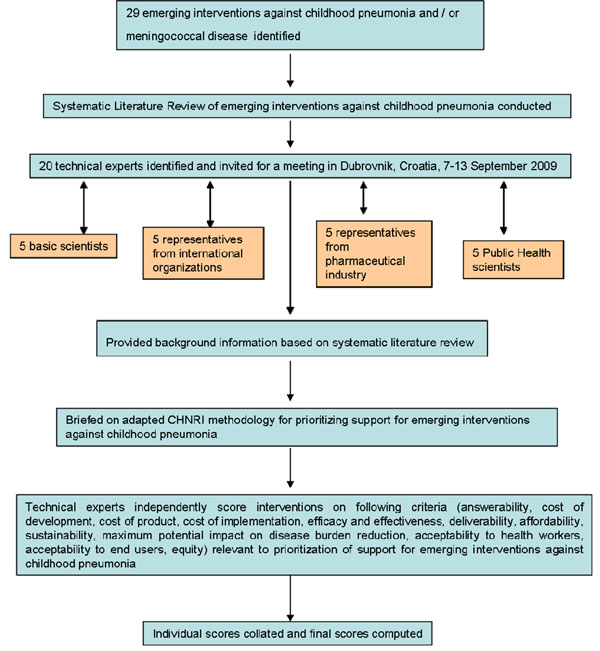
**A summary of Stage II of the CHNRI process of evaluation of an emerging intervention (an expert opinion exercise using the CHNRI criteria)**. CHNRI- Child Health and Nutrition Research Initiative

We shared the initial review of the literature with 20 experts. The list of chosen experts included five leading basic scientists, five international public health researchers, five international policy makers and five representatives of the pharmaceutical companies. The 20 experts were chosen based on their excellent track record in child health research (but were not specifically involved with measles disease research). We initially offered participation to the 20 experts with the greatest impact of publications in their area of expertise over the past 5 years (for basic researchers and international public health researchers), or for being affiliated to the largest pharmaceutical company in terms of vaccination programme or international agency in terms of their annual budget. For those who declined to participate (about 20%) replacements were found using the same criteria. The policy makers and industry representatives accepted our invitation on the condition of anonymity, due to sensitive nature of their involvement in such exercises. About half of the experts were either affiliated to institutions in developing countries or had previous experience of working in developing country settings. The experts met during September 7-13, 2009 in Dubrovnik, Croatia, to conduct the 2^nd^ stage of CHNRI expert opinion exercise. The process of second-stage CHNRI is shown in Figure 2. All invited experts discussed the evidence provided in CHNRI stage I, and then answered questions from CHNRI framework (see Supplementary table 2 in Additional file 1). Their answers could have been “Yes” (1 point), “No” (0 points), “Neither Yes nor No” (0.5 points) or “Don’t know” (blank). Their “collective optimism” towards each criterion was documented on a scale from 0 to 100%. The interpretation of this metric for each criterion is simple: it is calculated as the number of points that each evaluated type of emerging aerosolized measles vaccine received from 20 experts (based on their responses to questions from CHNRI framework), divided by the maximum possible number of points (if all answers from all experts are “Yes”) [13-17].

## Results

We identified 51 articles and product monographs for inclusion in the study. Currently several products are in development phase, some of which have completed phase I and II clinical trials.

### Answerabilty

We evaluated the likelihood of an effective aerosolised measles vaccine being available for incorporation into the routine Expanded Programme of Immunization (EPI) schedule or supplementary immunization campaigns within in a time frame of 10 years. The potential for measles aerosol vaccination has been recognized since the 1960’s when American and Japanese researchers acknowledged a consistent immunogenic response in children exposed to small amounts of aerosolized, partly attenuated measles vaccine [9]. Measles is an airborne disease, with transmission of the measles virus across the respiratory mucosa. Delivery of measles vaccination using aerosol technology aims to imitate this natural pattern of infection [24]. It is assumed that aerosol vaccination will generate immunity both locally in the respiratory mucosa as well as systemically. In Eastern Europe thousands of people have been immunized via the respiratory mucosa, with live attenuated vaccine against many infectious agents including anthrax and small-pox [25].

One of the formulations of the aerosolized measles vaccine proposes to use the same formulation as the existing subcutaneous measles vaccine i.e. aerosolize the liquid formulation of the current injectable vaccine. By adopting this strategy, MAP has managed to avoid years of investigations in attempting to reformulate the vaccine constitution [26]. Consequently, the development process is limited to creating an innovative device which can deliver the vaccine in a way that elicits an antibody response of equivalent or greater immunogenicity to that of the current vaccine.

Research efforts are also underway to produce an inhalable dry powder vaccine. In one of these formulations, the weakened measles virus will be mixed with high-pressure carbon dioxide to produce microscopic bubbles and droplets, which will then be gently dried to produce an inhalable powder [27].

The existing vaccine product is a viable selection for application to the aerosol device as it has a high success rate of provoking good seroresponse. Over ninety five percent of infants seroconvert if the first dose of the measles vaccine was given at 9 months, and over 99 percent seroconvert if the first dose was given at or after 12 months [5]. The question is whether this response is transferable to an aerosolized delivery system.

Two methods for the delivery of liquid aerosol vaccines are currently under trial. The inflatable bag aerosol method, a concept introduced in 2009, has only completed proof of principle trials and has not yet entered pre-clinical trials. The nebulized aerosol method has completed phase I clinical trials and will shortly be entering phase II/III clinical trials.

Robert Sievers and colleagues who have invented and patented a device known as Carbon dioxoide Assisted Nebuilization with a Bubble Dryer (CAN-BD) in a presentation at the 238th National Meeting of the American Chemical Society (ACS) indicated that the dry powder measles vaccine (developed in collaboration with Serum Institute of India) will be ready for entry into phase I clinical trials in India in 2010 [27]. They have also developed a dry powder live attenuated measles vaccine which does not require reconstitution and have demonstrated the safety and efficacy of the vaccine in rhesus macaques [28].

**Figure 3:**
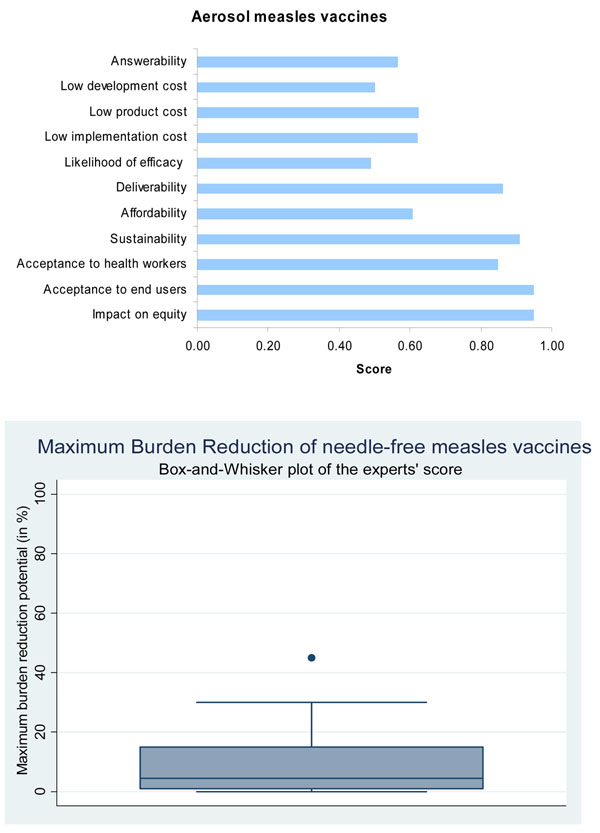
**The results of Stage II of the CHNRI process – an expert opinion exercise assessing the potential usefulness of investment in needle-free measles vaccines**. CHNRI- Child Health and Nutrition Research Initiative

Presented with these evidence, the panel of experts however expressed moderate levels of optimism (score around 60%) concerning the ability of aerosolized measles to satisfy the criterion of answerability (Figure 3).

### Efficacy and effectiveness

Liquid aerosolized measles vaccination has been evaluated in a multitude of field studies dating back over the last 30 years. The concept of an aerosolized vaccine was proven to be feasible in very early clinical trials carried out by Sabin et al. [29,30]. Following the results of these trials, several research groups, predominantly in Mexico and South Africa, pursued further research into measles aerosol vaccination. Results of major clinical and pre-clinical trials are outlined in Table 1 and Table 2 respectively.

**Table 1:** Summary of major clinical trials on aerosol measles vaccines presented to the expert group for stage II of the CHNRI process

Reference	MV Strain	Age group	Seroconversion as defined by authors
A.Dilraj et al [46]	MV-Schwarz ( s/c, aerosol), MV- Edmonston-Zagreb ( s/c, aerosol)	992 participants 5-14 years	Seroconversion rates (defined as four fold increase in antibody level): EZ Aerosol – 84.7% EZ sc – 78.8% SW Aerosol – 22.7% SW sc – 62.6%

A.Dilraj et al [47]	MV-Schwarz ( s/c), MV- Edmonston-Zagreb ( s/c, aerosol)	337 participants 5-14 years	Seroconversion (defined as four fold increase in antibody level) at 6 years after revaccination: EZ Aerosol – 86% EZ sc – 73% SW sc – 58%

J.A.Bellanti et al [48]	MV- Edmonston-Zagreb ( s/c, aerosol)	49 participants 6-7 years	The mean Specific anti-measles IgG antibody: EZ sc - 22.9 ± 4.6 EZ aerosol - 53.4 ± 9.4 Results are reported as mean _ µg/ml ± standard error of the mean

J.V.Bennett et al [49]	MV-Schwarz ( s/c), MV- Edmonston-Zagreb ( s/c, aerosol)	1624 participants 6-8 years	Change from seronegative to seropositive, >= 120 mIU/ml EZ aerosol – 65% EZ sc – 4% SW sc – 23%

R.M.Wong-Chew et al [50]	MV- Edmonston-Zagreb ( s/c, aerosol)	114 participants 11-13 months	Seroresponse rates as defined by four fold increase in antibody level: EZ aerosol – 89.8% EZ sc – 100%

R.M.Wong-Chew et al [51]	MV- Edmonston-Zagreb ( s/c, aerosol)	129 participants 8-10 months	Seroresponse rates as defined by four fold increase in antibody level: EZ Aerosol – 42% EZ sc – 67%

**Table 2:** Summary of major pre-clinical trial studies of aerosol measles vaccines presented to the expert group for stage II of the CHNRI process

Reference	Factor for investigation	Results	Comment
Low, N et al de Vries et al [24,35]	Vaccine strain	EZ > Schwarz in all studies.	In South Africa, the Schwarz strain was inactivated as an aerosol.

Laube, B.L [26]	Device selection	3 systems for entry at clinical trial: Un-vented, Breath-enhanced & Ultrasonic nebulizer. All models fulfil safety and logistic criteria.	Suitability criteria – portable, easy to use, battery-operated, sanitary, operable with replacement parts.

Coates, A.L et al[52]	Viral particle size	Minimum < 10μm Deep lung deposition <5μm	Size of inhaled droplet is best determinant of lung deposition

Coates, A.L et al[52]	Number of infective particles	<1000 pfu’s; 30-250 pfu’s required to stimulate immune response	5000 pfu’s delivered in percutaneous measles vaccine

Cohen, B.J et al[53]	Potency retention	85-102% vaccine potency retention in all 3 measles aerosol device’s.	Potential disaggregation of viral particles during aerosolization accounts for results >100%

de Swart, R. L et al [54]	Animal model – safety	No superimposed risk in immunocompromised or asthmatics. No risk of vaccine-associated encephalitis or Bells palsy identified.	Concern of illness exacerbation in vulnerable groups. Postulation of direct CNS contact through cribiform plate precipitating neurological symptoms.

Low and colleagues in a meta-analysis of results from 7 randomized trials, 4 non randomized trials, and 6 uncontrolled studies demonstrated that in the case of children aged below 10 months, subcutaneous measles vaccine showed a greater seroconversion rate compared to those using the aerosol route [24]. They suggested that this was perhaps partly due to persistent maternal antibodies (at this age) that interfered with the immune response and partly due to the poor suitability of the aerosol device for this age group. However, in children aged 10 months and older, aerosolized measles vaccine was found to be more immunogenic than the subcutaneous one. An earlier systematic review and meta-analysis by Hiremath and Omer in 2005 using data from 20 results from 16 studies concluded that the measles vaccine administered via respiratory route is as at least as immunogenic as subcutaneous vaccine [31]. Questions however remain regarding the optimal age for the first dose of the vaccine in high burden areas and the optimal number of doses required to achieve a sero-conversion rate above 95 percent in the population.

**Figure 4:**
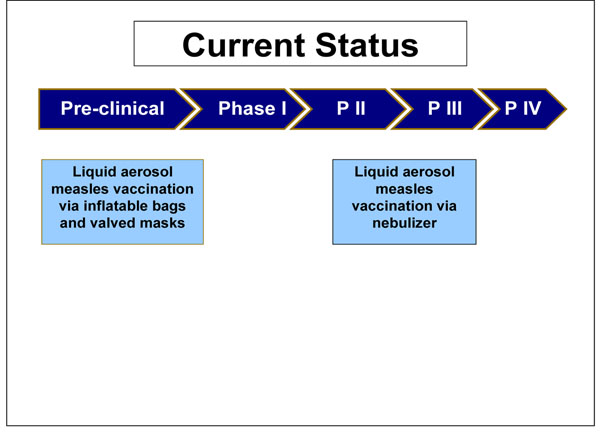
The current status of the research into measles aerosol vaccines presented to the expert group for stage II of the CHNRI process

The nebulised aerosol measles vaccination entered stage I clinical trials in India in 2006. There, 60 subjects were assessed for their immunological responses at 3 different sites. The trial was due for completion in 2008, but no results have yet been published. Preliminary data suggest a low adverse event profile and good provocation of immunogenecity. Late stage clinical trials were due to commence in 2007, but at present there are no published data to ascertain the progress of the vaccine (Figure 4).

A recent “proof of principle” trial has been completed using inflatable bag and valved mask technology. The bags with 3-4 litre capacity were inflated with 0.053 ml of vaccine in roughly 4 seconds, and inhaled until total collapse of the bag was seen. Data from this trial demonstrated almost double the number of children showing a 4-fold or 2-fold boosted antibody response when vaccinated via the inflatable bag than by injection. Though this model proposes to offer similar advantages to the nebulised formulation, it should be able to deliver a more measurable dose between individuals and hopes to offer greater protection in infants (Figure 4).

**Figure 5:**
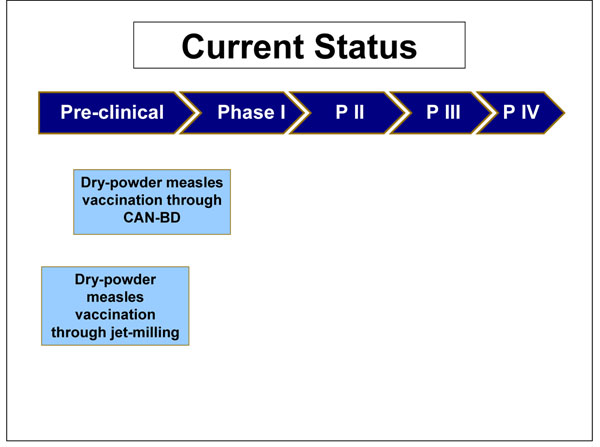
The current status of the research into measles dry powder product presented to the expert group for stage II of the CHNRI process

The dry powder vaccination has had a more protracted course of development (Figure 5). Although the limited studies on such formulation have all highlighted the plausibility of a marketable dry powder product, none of the published data through May 2009 showed a specific in vivo demonstration of clinical safety and efficacy (Table 3). Scientists in the United States have recently demonstrated that respiratory delivery of a single dose of dry powder live attenuated measles vaccine in rhesus macaques is capable of inducing durable fully protective immunity comparable to the injectable vaccine [28].

**Table 3:** Summary of studies of dry powder measles product presented to the expert group for stage II of the CHNRI process

Reference	Factor for investigation	Results	Conclusions
LiCalsi, C., et al [55]	Feasibility of dry powder inhalation in measles	Optimal vaccine delivery site – lungs; Particle sizing 1-5μm; Preparation – micronization and jet-milling ;3 Spiros delivery devices designed	Undemonstrable clinical application

de Swart, R.L., et al [56]	Dry powder vaccination in Macaques	Low seroresponse to measles dry powder blend compared to injection or liquid aerosol vaccination	Proof of principle evident by stimulation of weak immune response. Poor device design in macaque model – loss of vaccine at delivery.

LiCalsi, C., et al [57]	Dry powder measles vaccine potency retention	Up to 89% viral potency retention can be achieved with micronization.	

Burger, J.L., et al [58]	Stabilizing dry powder measles formulations	Myo-inositol> trehalose as a sugar stabilizer in dry powder measles vaccinations	Myo-inositol is relatively unhygroscopic, improving its dry powder vaccination credentials

Presented with the evidence from the literature through May 2009 (Table 3), the panel of experts expressed concern (score around 50%) regarding the ability of aerosolized measles vaccines to satisfy the criterion of efficacy (Figure 3).

### Deliverability, affordability and sustainability

Deliverability refers to the prospects of an emerging intervention to overcome environmental and economical barriers to achieve high coverage, particularly in low resource settings. This criterion takes into account the level of difficulty with aerosolized measles vaccine delivery, the infrastructure and other resources available to implement the intervention and also government capacity and partnership requirements for achieving near-universal coverage with this new intervention. The aim of MAP (as well as other aerosol measles vaccine efforts) is also to develop a product that is primarily suitable for use in low resource environments, and is capable of quick implementation in mass campaigns.

#### Intervention

The existing subcutaneous measles vaccine is associated with some safety risks. In the developing world unsafe needle practices put individuals at the risk of blood borne viral transmission, including hepatitis C virus (HCV), hepatitis B virus (HBV) and human immunodeficiency virus (HIV) [32]. Unsafe injection practices comprise needle re-use, inefficient needle sterilization and improper needle waste disposal. In addition, needle-stick injuries within the healthcare profession are not infrequent, carrying an equivalent risk of blood-borne virus transmission. Approximately 3 million health workers across the globe are injured by sharps contaminated with HBV, HCV or HIV annually [32]. The nebulized device is designed to be easily administered. This should lower training requirements for individuals administering the measles vaccine [33]. Consequently, this should enable a wider span of vaccine delivery, with the potential to employ non-healthcare staff within rural communities to distribute and deliver the vaccine. This would significantly ease pressures on under-staffed health care systems, such as most of those in the developing world. The oral polio vaccination campaign is one good example of such vaccination strategies implemented using non-medical personnel [34]. Furthermore, this would reduce the overall cost of the program. However, a standard dose of aerosolised vaccine is inhaled over 30 seconds with minimal preparation time [35]. Because of this, an increased health worker contact time is anticipated. This may result in lesser number of vaccinations delivered each day, which could undermine mass vaccination campaigns when very large numbers of children need to be immunized.

#### Expanded program on immunization

The new intervention would fit into the current Expanded Program on Immunization (EPI) schedule. Guidelines for measles vaccination of infants at 9 months exist in countries with high disease burden for measles. In countries where measles vaccine uptake is high, children are routinely immunized between 9 and 12 months [36]. The Working Group on Measles (WGM) recommends a second measles dose (MCV2) in countries which achieve over 75% vaccine coverage of MCV1. SIAs are encouraged every 2-4 years, used as either follow-up or catch-up programmes, targeting children born since the last campaign. They allow for children, who have missed their measles vaccination through the national immunization schedule, to have another opportunity for vaccination. From 2000 to 2007, 576 million children between 9 months and 14 years of age were vaccinated against measles through SIAs [4].

#### Infrastructure

Cold chain has a limited coverage and integrity in developing countries, resulting in restrictions on vaccine distribution [37]. The existing measles vaccine is relatively thermo stable prior to reconstitution[38]. After reconstitution, the vaccine must be discarded of within 6 hours due to risks of potency loss and contamination. Heat damage occurs through interruptions in the cold chain or heat shock to the vaccine from the addition of a warm diluent [38].

The liquid aerosol measles vaccine demands no alteration to the existing measles cold chain requirements. However, more space may be required to store the delivery device. Limited storage space is an issue in many developing countries. The dry powder formulation proposes to eliminate cold chain requirements. The potency of the dried vaccine was tested and the results showed that the dry-powdered aerosol was stable for at least eight weeks at 37°C [27]. Cold chain facilities maintenance costs are approximately $200-300 million annually [32] and are responsible for almost one third of the UNICEF’s annual budget for immunization [39]. If research could be directed at producing more vaccines that did not require cold chain, vast savings could be made. Additionally, without the limitations of cold chain, the vaccine could be stored for expansive lengths of time without degeneration and could be further distributed to remote areas with no cold chain facilities.Based on these evidence, the panel of experts expressed high levels of optimism (score around 80%) regarding the deliverability of the aerosolized measles vaccine (Figure 3).

### Cost

MAP receives financial and technical support from several agencies, most notably the Bill and Melinda Gates Foundation, the American Red Cross, the WHO and the US Centers for Disease Control and Prevention. Generally, measles is a relatively under-funded disease for which the funding gap stood at $176 million for the year 2009-2010 [4]. The existing measles delivery system is affordably priced at $0.06 per dose. While the aerosol device itself is more expensive, savings are expected to be noticed in the returns gained from diminished blood borne virus transmission. A recent cost-analysis model estimated that the societal cost of inappropriate needle use leading to blood borne virus transmission totals $26.77 per injection [40]. It is presumed that, based on this model, there will be cost-savings of an aerosol device when the wider connotations of the current vaccine are factored in. A review assessing the potential economic impact of introducing a thermo-stable vaccine in Cambodia, Bangladesh and Ghana predicted its high cost-effectiveness in all three countries [41]. Savings would be made through reduced vaccine wastage, the avoidance of adverse events from unsafe needle practice, as well as savings from the subtracted cold chain requirements [32]. Presented with these evidence, the panel of experts expressed concerns (score around 50%) over the ability to develop a low-cost aerosolized vaccine. However, given the very high level of interest in the MAP from various funding agencies, the group felt moderately optimistic (score around 60 percent) regarding possiblity to make the vaccine available at a lower price to the consumers in developing countries. They also were moderately optimistic (score around 60 percent) that the production and implementation cost of the vaccine could be kept low. The low pricing should also be sustainable, given the high level of commitment of many donors towards measles elimination programme (Figure 3).

#### Local and context-specific factors

Health systems of all developing nations are not homogeneous and therefore cannot be expected to show exactly the same response in regards to all factors related to deliverability of any health intervention [42]. Unstable political environment, civil war and natural disasters are examples of circumstances which can drastically interfere with the distribution of even the most cost-effective healthcare strategies. Measles control in the Eastern Mediterranean region has been plagued with slow progress due to turbulent political climate in a number of countries [4]. The deliverability factors discussed here would have their highest index of impact only if a strong political leadership and commitment could be generated.

### Maximum potential for disease burden reduction

“Disease burden reduction” considers whether there will be a significant percentage decrease in global measles mortality (mainly due to the complication of severe pneumonia) with the introduction of the newly proposed interventions. In 2008, approximately 164,000 individuals died as a consequence of measles despite considerable efforts to prevent and control disease outbreaks. Over 90% of these deaths were estimated to have been in children aged below 5 years [4]. Urbanization and rapid population growth pose special challenges for measles eradication since they facilitate person-to-person spread of the highly contagious measles virus; experience shows that these conditions require sustained immunization coverage levels of >95% to interrupt transmission. The WHO region of Americas has eliminated measles transmission using the supplementatary immunization activities targeting children aged 9 months to 14 years to quickly raise immunization coverage to ≥95 percent [5]. In 2007, 65% of children not receiving their first dose of measles vaccine came from only 8 countries: India, Nigeria, China, Ethiopia, Indonesia, Pakistan, the Democratic Republic of Congo and Bangladesh [7]. This demonstrates that the barrier to elimination and eradication of measles is not in the vaccine per se but in its delivery. Hence, greater investments in either improvement of existing intervention strategies to increase coverage or identifying new delivery mechanisms are needed [5,43].

A study of vaccination in Mongolia indicated that vaccines that reach rural communities suffer increased breaks in the cold chain, exposing the vaccine to unsuitable temperatures. Consequentially, rural children exhibited lower antibody response than urban children [44]. With the dry powder measles vaccine formulation, the cold chain requirements can be obviated and therefore there is a possibility for even wider immunization coverage by improved access to marginalised communities and those residing in remote and hard to reach areas resulting in greater decline in disease burden. Based on these evidence and their experience in developing countries, the panel of experts felt that the aerosolized measles vaccine would have modest levels of maximum impact on overall pneumonia disease burden (median: 5%, interquartile range 1-15%, minimum 0%, maximum 45%) when compared to the existing measles vaccine (Figure 3).

### Equity

The assessment of a vaccine according to “equitability” takes into account the predicted effects that implementation of the new technology will have on poor populations within countries. The score is high when the experts agree that the resultant impact will reduce health inequities between rich and poor social groups. The highest impact of measles mortality is on the poorest communities in the world [7]. The aim of introducing a novel intervention is to target this section of society in order to decrease health inequities. In Latin America, measles has been the target of a large scale intervention programme to try and eliminate the disease [43]. In a region with very large disparities in economic wealth, an equitable response to the programme was achieved by all nations, demonstrating that the “...*successful implementation of immunization strategies is more important to achieve elimination than the under-lying socio-demographic circumstances of the country*” [36].

The greatest uptake of an intervention is amongst the highest social groups, with the lowest social classes suffering from diminished intervention coverage, weaker health services and greater disease exposure. This is liable to continue to be the case for any new intervention which does not aim to tackle the distributive factors, and which fits the same format as the existing technologies. In the case of novel interventions, such as the measles aerosol vaccine, there is the potential to counteract this monotonous pattern of uptake especially using SIAs which target the vulnerable and marginalised communities in developing countries. Disparities in the coverage of immunization between the rich and poor are reduced relative to other interventions. This suggests that investing in immunization as a health strategy is advantageous as the uptake across the spectrum of society is close to indifferent and therefore there will be a reduced gradient of health inequity [45]. The panel expressed very high levels of optimism (score over 90%) regarding the ability of the aerosolized measles vaccine to have a positive impact on equity (Figure 3).

### Acceptability to end users

Mothers claimed to prefer the needle-free route in early measles aerosol clinical trials held in Mexico [11]. Pain and phobic associations with needle-syringe delivery systems are the main limiting factors and they result in reduced compliance with injectable measles vaccine. Since the attitudes of parents (with regard to injectable vaccines) are largely similar across the globe, it may not be incorrect to generalise the results. However, community based trials or post licensure field effectiveness trials will be needed to assess the safety and acceptability of aerosol based vaccines more formally. The experts were very optimistic (score over 80%) that the vaccine would be acceptable to both the end-users and health workers (Figure 3).

## Discussion

Measles is an important risk factor for childhood pneumonia and thus is responsible for a large proportion of pneumonia associated mortality in young children especially in low and middle-income countries where the disease burden due to measles continues to be high. Although an effective measles vaccine exists, there are operational challenges concerning the delivery of the vaccine to achieve universal coverage. The literature review summarized in this paper presents the evidence required for making an informed decision on the research priority that should be given to aerosolized measles vaccine to reduce the burden of childhood pneumonia. The scores for the liquid and dry powder formulations of the inhalable measles vaccine against the set criteria represent the collective optimism of a panel of experts drawn from varying technical backgrounds and affiliations. Although there is currently no licensed aerosolized measles vaccine, significant progress is being made for developing one such vaccine.

Intensification of the measles vaccination programme over the past decade has helped to dramatically reduce the global disease burden due to measles and thus the childhood pneumonia mortality. However, the rate of decline in measles mortality is unlikely to continue at the same level for long. In order to sustain and enhance the gains made in reducing the disease burden associated with measles, some new approaches are needed. In Latin America, scaling up of existing vaccination services in order to decrease the number of measles cases across the region has been a very successful strategy [4]. This case of strengthening the investments in existing vaccination programmes should be acknowledged as a prospective strategy to be applied in other regions of the world.

However, investment in novel vaccinations may offer a promising mode of challenging currently limiting deliverability factors. In the case of candidate aerosolized measles vaccines, the experts expressed low levels of optimism regarding the criteria of likelihood of efficacy, low cost of development and modest impact on reducing the burden of childhood pneumonia; moderate levels of optimism regarding answerability, low cost of production, low cost of implementation and affordability; and high levels of optimism regarding deliverability, sustainability, impact on equity and acceptability to health workers and end-users. Further research is needed to establish the safety and optimal age for primary vaccination using aersolized measles vaccine. The liquid aerosol vaccination needs to undergo more trials in children aged less than 5 years as the majority of immunization successes are demonstrated in older children. Efficient post-licensure surveillance systems need to be put in place to monitor coverage and ensure reporting of adverse that adverse following immunization. For an optimal dry powder measles vaccination product, it is important that along with assessment of safety and effectiveness of the vaccine, the maximal device outputs be calculated before entry into the clinical trial process.

This is the first time such an exercise has been conducted with the aim of predicting the future impact of emerging vaccines on childhood pneumonia. The CHNRI methodology was primarily designed to evaluate existing interventions and competing investment priorities for health research. Even though we used the CHNRI criteria, we modified it by including systematic review of available literature and not involving all stakeholders (e.g. end-users and health workers). The scores reported in this paper express the collective opinion of a panel of 20 experts. Although this could be pointed out as a limitation, the main strengths of this approach are that it aims to be objective, systematic, evidence based and explicit.

## Conclusions

To summarize, aerosolized measles vaccine presents an unique opportunity to not only control and eliminate measles morbidity and mortality in young children, but also decrease the overall burden of disease due to severe pneumonia in young children. Although there has been considerable progress in achieving the vision of effectively delivering measles vaccine through the respiratory route, it will still be a few years before such a vaccine is ready to be incorporated into the routine EPI programmes in high disease burden areas.

## Competing interests

DH, ET, HN, TH, LZ, SBO, IR and HC declare that they have no competing interests. SSJ is employed by Serum Institute of India Ltd. which has co-developed the liquid and dry powder aerosol measles vaccines.

## Authors’ contributions

DH, HN and TH were responsible for the acquisition of the data and conducted the literature review. ET, HN and LZ helped design the study’s analytic strategy and prepared the Materials and Methods, Results and Discussion sections of the text. SSJ and SBO critically reviewed and revised the manuscript for important intellectual content. HN carried out the final revisions. HC and IR designed the study and provided guidance in analysis and writing of the manuscript. All authors read and approved the final manuscript.

## Supplementary Material

Additional file 1 Supplementary tables: Supplementary Table 1: Details of search strategy for identifying studies reporting aerosolised measles vaccine Supplementary Table 2: Questions used in the Phase II CHNRI process Click here for file
